# Role of Rab10 in cocaine-induced behavioral effects is associated with GABAB receptor membrane expression in the nucleus accumbens

**DOI:** 10.3389/fphar.2024.1496657

**Published:** 2024-11-27

**Authors:** Zhuoxuan Yu, Qiang Fu, Tianyun Qiu, Caidi Yang, Mingfen Lu, Qinghua Peng, Jianhua Yang, Zhenzhen Hu

**Affiliations:** ^1^ The First Clinical Medical College, Jiangxi Medical College, Nanchang University, Nanchang, Jiangxi, China; ^2^ Department of Respiration, Department Two, Jiangxi Provincial People’s Hospital, Nanchang, Jiangxi, China; ^3^ Department of Clinical Laboratory, Wuhan Hankou Hospital, Wuhan, Hubei, China; ^4^ Department of Pathophysiology, School of Basic Medical Sciences, Jiangxi Medical College, Nanchang University, Nanchang, Jiangxi, China; ^5^ Department of Anesthesiology, The 1st Affiliated Hospital, Jiangxi Medical College, Nanchang University, Nanchang, Jiangxi, China; ^6^ Department of Physiology, School of Basic Medical Sciences, Jiangxi Medical College, Nanchang University, Nanchang, Jiangxi, China

**Keywords:** cocaine, nucleus accumbens, behavioral sensitization, ras-related GTP-binding protein Rab10, gamma aminobutyric acid type B receptor

## Abstract

**Aim:**

Previous studies have demonstrated that Ras-related GTP-binding protein Rab10 (Rab10) plays a role in psychostimulant-induced behavioral effects. In this study, we showed that Rab10 in the nucleus accumbens (NAc) of male animals affects the development of cocaine-induced behavioral effects, which are associated with the plasma membrane expression of the GABA_B_ heteroreceptor (GABA_B_R).

**Methods:**

We performed flow cytometry, immunoendocytosis, pHluorin activity analysis, electrophysiology analysis, and open-field testing to explore the role of Rab10 in modulating the membrane expression and function of GABA_B_R and its regulatory effect on cocaine-induced behavioral effects.

**Results:**

Transcriptomics analysis showed that *Rab10* was elevated following acute cocaine treatment. Membrane levels of Rab10 increased within day 1 of the cocaine treatment, subsequently decreasing at later time points. *Rab10* deficiency in NAc regions significantly increased cocaine-inhibited membrane GABA_B_R levels and inhibited cocaine-induced hyperlocomotion and behavioral sensitization. In addition, *GAD*_*67*_^*+*^-expressing neurons from NAc regions treated with cocaine revealed a significant decrease in Rab10 membrane expression. Furthermore, NAc neuron-specific *Rab10* knockout resulted in a significant increase in the cocaine-inhibited membrane expression of GABA_B_R, along with increased miniature inhibitory postsynaptic current (mIPSC) amplitude and attenuation of baclofen-amplified Ca^2+^ influx.

**Conclusion:**

These results uncover a new mechanism in which Rab10-GABA_B_R signaling may serve as a potential pathway for regulating cocaine-induced behavioral effects.

## 1 Introduction

A previous study showed that Ras-related GTP-binding protein Rab10 (Rab10) in the nucleus accumbens (NAc) region is a critical target for psychostimulant drug-associated behavioral changes, as demonstrated by proteomics analyses and open-field tests (OFTs) (Vanderwerf et al., 2015). Rab10 is extensively expressed in brain regions such as the cortex and striatum, where it is present in most neurons and glial cells (Zhang et al., 2016b). Morphologically, Rab10 is associated with the trans-Golgi network (Liu and Storrie, 2012), endosomes, (Chen et al., 2006) and plasmalemmal precursor vesicles (Xu et al., 2014), all of which modulate membrane trafficking events (Chua and Tang, 2018). Accumulating evidence shows that modulating the activity of Rab10 will alter the balance of receptors between the plasma membrane and endosomal residential pool (Xu et al., 2023). Previous research has reported that Rab10 regulates the cell surface delivery of the δ-opioid receptor (DOPr) (Degrandmaison et al., 2020), as well as Toll-like receptor 4 (Wang et al., 2010) and glucose transporter 4 (Brewer et al., 2016). Furthermore, Rab10 also plays a role in neurodegenerative diseases such as Alzheimer’s disease (Ridge et al., 2017). However, the link between Rab10 deficits and the associated receptor’s membrane expression and function in the context of cocaine addiction remains unclear.

In the central nervous system, abnormal GABA_B_ heteroreceptor (GABA_B_R) signaling and membrane stability have been linked to an array of psychiatric disorders, such as drug addiction (Padgett et al., 2012) and epilepsy (Fritzius and Bettler, 2020). GABA_B_R primarily regulates the inhibitory pathway through plasma membrane stability and availability. GABA_B_R is a critical target for therapeutic agents, including addictive psychostimulants such as amphetamine and cocaine, which inhibit GABA_B_R function (Stafford et al., 2020; DeBaker et al., 2021). Previous clinical and preclinical research indicates that a selective GABA_B_R agonist, baclofen, attenuates addiction behaviors, including drug-seeking behavior and drug craving (Kent et al., 2020), while decreased GABA_B_R expression augments the response to cocaine-induced locomotor activity (DeBaker et al., 2021). Baclofen is reported to amplify Ca^2+^ entry through voltage-dependent R-type Ca^2+^ channels, thus reducing conditioned fear (Zhang et al., 2016a). Nevertheless, the molecular and cellular mechanisms that regulate the membrane expression and the function of GABA_B_R on NAc neurons in cocaine addiction are still largely unexplored.

In this research, repeated-cocaine treatment decreased the membrane expression of Rab10 in NAc regions. Furthermore, genetic ablation of *Rab10*, specifically in NAc neurons, inhibited the membrane expression of GABA_B_R and reduced the frequency of miniature inhibitory postsynaptic currents (mIPSCs) but did not affect locomotor activity under normal physiological conditions. However, repeated cocaine treatment resulted in the opposite effect, i.e., a significant increase in the membrane expression of GABA_B_R and mIPSC amplitude and significant inhibition of baclofen-amplified Ca^2+^ influx and cocaine-induced behavioral effects. This study highlights the crucial role of Rab10 in regulating GABA_B_R membrane expression during cocaine-induced behavioral effects.

## 2 Materials and methods

### 2.1 Animals

Experiments were conducted on male Sprague–Dawley (SD) rats, with an average weight of 250 g. This study was approved by the Ethics Committee for Animal Experiments at the University of Nanchang (RRID: RGD_728193; Permit Number: 2010–0002). As is common with most other experiments on cocaine addiction, we excluded female rats to ensure stability in the results of behavioral testing. All experimental procedures adhered to the US National Institutes of Health Guide for the Care and Use of Laboratory Animals. Efforts were made to alleviate discomfort, suffering, and pain in experimental animals. Rats that were not within the age range of 2-months ± 2 weeks were excluded. The *nestin-Cre* mice have been introduced in prior publications (Tronche et al., 1999). With loxP sites flanking exons 4 and 5 of the *Rab10* gene, *Rab10* ^Floxed/Floxed^ (*Rab10* ^F/F^) mice were utilized in this study (Biocytogen Company, Beijing, China). The primers used for genotyping conditional knockout mice are listed as follows: *cre*, 5′-TCG​ATG​CAA​CGA​GTG​ATG​AG-3’ (P1) and 5’-TCC​ATG​AGT​GAA​CGA​ACC​TG-3’ (P2); *Rab10,* 5′-CAA​CCT​AAT​CAT​ACT​AAT TAGATTGGT-3’ (P3) and 5′-GAT​GCC​TAT​TGT​TAG​TGA​TAC​TAC-3’ (P4). All mice involved in this study were on a *C57BL/6J* background. *GAD*_*67*_-GFP knock-in mice *(GAD*_*67*_^*+*^ mice) were generously provided by Dr Shujia Zhu (Center for Excellence in Brain Science and Intelligence Technology, Chinese Academy of Sciences, Shanghai 200031, China).

The experimental procedures within this study adhered to pseudo-randomized and repetitive-measurement rules. For scientific grouping, a computer-based random number generator was employed. The *in vivo* experiments, involving six rats per group, were repeated three times. 1) In three repeated experiments, 36 male SD rats were randomly assigned to two groups (cocaine and saline, 15 mg/kg), with each group comprising six rats. 2) The AAV-GFP or the AAV-Rab10-siRNA-GFP construct was injected into the bilateral NAc of 72 rats. Subsequently, these rats were injected with either cocaine or saline (15 mg/kg, i.p.). Based on different treatments, these rats were randomly allocated into four groups, with each group comprising six rats. No animals were sacrificed during the experiments.

### 2.2 Gene expression analysis

Gene expression profiles from an independent dataset were downloaded from the Gene Expression Omnibus (GEO). The dataset (Accession Number: GSE18751) was used to investigate the differential expression levels of candidate genes of interest and perform pathway enrichment analysis. Collected using the Illumina MouseWG-6 v2.0 Expression Beadchip platform, this dataset consists of 12 whole-genome expression profiles of NAc samples from C57BL/6J mice treated with acute cocaine (20 mg/kg, i.p.) or saline. Based on the *Rab10* expression levels, two groups of samples were selected to perform differential expression analysis. The top quartile was defined as the *Rab10* high-expression group, and the bottom quartile was defined as the low-expression group. Genes with a false discovery rate (FDR) of *p* < 0.05 and |fold change| > 1.5 were considered highly differentially expressed. Pathway enrichment analysis was carried out using the DAVID tool (version 6.8), and the R package limma was used to perform differential expression analysis.

### 2.3 Reagents and antibodies

Antibodies used in this study were obtained from the following companies: Proteintech (Rab10-11808-1-AP for Western blot and immunostaining); R&D Systems (GABA_B2_R-AF1188 for Western blot and immunostaining); Sigma (pan-Cadherin-C1821 for Western blot); and Millipore (GABA_B1_R-MABN492 for Western blot and immunostaining). DAPI (4′,6-diamidino-2-phenylindole) was procured from Beyotime Biotechnology. For immunostaining, the secondary antibodies were obtained from Invitrogen. Horseradish peroxidase-conjugated secondary antibodies were obtained from Millipore. In this work, GABA_B1_R-pHluorin constructs were developed. GCaMP6 and MYC-GABA_B1_R were provided by Dr Shujia Zhu (Center for Excellence in Brain Science and Intelligence Technology, Chinese Academy of Sciences, Shanghai 200031, China).

### 2.4 Membrane protein extraction

The cells or tissues of NAc were homogenized in lysis buffer that includes 1% Nonidet P-40, 50 mM Tris-HCl, pH 7.5, 0.5% sodium deoxycholate, 150 mM NaCl, and protease inhibitors (Cocktail set III, 539134, Merck/Millipore). Membrane proteins of cultured primary neurons or NAc brain regions of rats were prepared using the membrane protein extraction kit (k268-50, BioVision), followed by immunoblotting experiments using specific antibodies, as detailed in the *Reagents and Antibodies* section.

### 2.5 Western blot analysis

The protein was separated on a 10% SDS-PAGE gel and subsequently transferred onto a polyvinylidene fluoride (PVDF) membrane. The membrane was subsequently blocked in Tris-buffered saline containing 5% skimmed milk powder for 2 h at room temperature, followed by incubation at 4°C with the primary antibodies at the desired concentration overnight. Primary antibodies used were as follows: Rab10 (1:1,000), pan-Cadherin (1:1,000), GABA_B1_R (1:1,000), and GABA_B2_R (1:1,000). Thereafter, the membrane was incubated with appropriate secondary antibodies coupled with HRP for 2 h at room temperature. The bands’ chemiluminescence signals on the film were detected using a chemiluminescence system, and the intensity of these bands was quantified using ImageJ software.

### 2.6 Surgery and intra-nucleus accumbal infusion

Our preliminary experiments (Meng et al., 2018), along with a previous study (Sano et al., 2016), demonstrated that sodium pentobarbital functions as a short-acting anesthetic that exerts fewer side effects on the blood–brain barrier and cardiovascular function than those observed with other anesthetics, such as isoflurane and ketamine/xylazine. Rats were thus anesthetized prior to surgery with sodium pentobarbital (dissolved in saline, 42 mg/kg, i.p.; Sigma Co., St. Louis, MO). Placed in the stereotaxic frame (Neurostar Co., Sindelfingen, Germany), rats were surgically implanted with bilateral stainless-steel guide cannula assemblies (22 gauge; Plastics One, Roanoke, VA) directly into the regions of bilateral NAc shells during an aseptic stereotaxic procedure. The guide cannula was positioned using stereotaxic coordinates relative to bregma: M/L+ 1.6 mm, A/P +1.7 mm, and D/V −7.5 mm. Using dental cement and two fixed small stainless-steel screws, the guide cannula assemblies were securely affixed to the skull. The screws were carefully inserted to a deliberate shallow depth to prevent potential damage to the brain. A dummy cannula was positioned to prevent blockage. A dust cap was also screwed onto the top of the cannula assemblies. Following the surgical procedure, rats were placed in a heated chamber for approximately 1 h to recover from the anesthesia.

The health condition of the rats was closely monitored after surgery. Prior to the start of the experiments, at least 10 days of recovery was allowed. To perform the infusions, stainless-steel injector cannulas (28 gauge; Plastics One) fixed to 5-μL syringes (Neurostar Co., Sindelfingen, Germany) and polyethylene-10 tubing were used. Viral supernatant (1 μL/side) was injected into the bilateral NAc shells over a period of 120 s. Following the completion of the infusion, the injection needle was maintained in place for 10 min.

### 2.7 Adeno-associated virus production and transfection

Constructed and encapsulated by Shanghai GeneChem Co., Ltd, replication-deficient adeno-associated virus (AAV) carrying the gene for Rab10 was used in this study. The mean titer of the viral stocks, derived from transfected HEK 293 cells, was measured at 7.0 × 10^9^ infectious units/ml. For viral injection, 1 µL of the concentrated AAV solution was injected into the rat NAc shell. Polybrene (5 μg/mL) was included in the viral solutions to improve infection.

### 2.8 Measurement of locomotor activity

One day before experiments, rats were introduced to an activity cage (100 × 100 × 40 cm) for a 30-min habituation. Right after the injection of cocaine (15 mg/kg, i.p.), the locomotor activity of the rats was assessed in activity cages (Panlab Harvard Apparatus, Spain). Open-field tests (OFTs) were conducted to assess locomotor activity for 7 successive days after treatment. Cameras were fitted right above each cage and linked to a computer that had SMART software installed (version 3.0.01, Panlab S.L.U). Rats were allowed to perambulate in the activity cage under illuminated conditions for 10 min. As rats broke the infrared beam path, their movement was tracked, and the connected computer recorded the total distance traveled.

### 2.9 NAc cell culture

Adhering to the National Academy of Sciences’ guidelines for the care and use of laboratory animals, adult pregnant female mice were euthanized by asphyxiation in a CO_2_ chamber. Consistent with prior research, NAc cells harvested from the brains of embryonic day 18 mice were rinsed in cold HBSS (Shi and Rayport, 1994). Incubation of the cells in 2 mL HBSS was followed by their dissociation using 2 mL of trypsin-EDTA (Sigma) for 12 min at 37°C. To terminate the dissociation, 2 mL of inoculation medium was applied, containing neurobasal growth media, 1% B27, 5% fetal bovine serum (FBS), and 1% glutamic acid. A 5-min centrifugation process was performed at 1,000 r/min. For plating, NAc cells were adjusted to a density ranging from 10^5^ to 10^6^ in the growth medium. The growth medium, which contains the components neurobasal growth media, 1% B27, and 1% glutamic acid, was replaced every 3 days. Cells were utilized after 14 days of growth.

### 2.10 Immunofluorescence and image processing

Derived from *GAD67* ^*+*^ mice at embryonic day 18, 48 NAc cultures were measured after exposure to different treatments: 1 μM cocaine for 5 or 10 min, 100 μM baclofen for 30 min, and saline. Each group consisted of four cultures and was replicated three times. The 48 NAc cultures obtained from wild-type (WT, Nestin^+^; *Rab10* ^+/+^) and *Rab10*-deficient (Nestin^+^; *Rab10* ^F/F^) mice underwent measurement subsequent to saline or cocaine (1 μM, 5 min post-treatment) treatment, with four cultures per group across three replicates. For membrane immunofluorescent labeling, live neurons were subjected to 15-min incubation at 37°C with antibodies against GABA_B1_R, GABA_B2_R, and Rab10. After being exposed to cocaine or baclofen at 37°C, the cell slices underwent a 10-min fixation in 4% paraformaldehyde in PBS, pH 7.4 with no permeabilization. Next, they were washed twice in PBS for 5 min. Subsequently, a 10-min incubation at room temperature was applied to the cell slices using a 3% solution of hydrogen peroxide (H_2_O_2_), followed by two rounds of washing in PBS for 5 min each. Fluorescence-conjugated secondary antibodies (Alexa 647 and Alexa 568, 1:400, Invitrogen) were applied in an appropriate amount at room temperature for 120 min, followed by 5 min of PBS washing.

To determine the fluorescence intensity of the membrane, confocal microscopy was utilized with a ×20 objective. Pinhole, gain, and laser were set to the same levels across all images. Using a NIKON TiE or A1R Laser Scanning Confocal Microscope, 24 images were captured from the NAc cell cultures of three different animals, all at an identical focal level. Captured at 1-μm intervals, images were reconstructed into three dimensions (3D) that incorporated 40 to 50 Z-stacks. The receptors’ membrane fluorescence images were captured from the monolayer cell membrane at peak intensity, and the images were then processed using Fiji.

### 2.11 Flow cytometry analysis

Rab10 membrane expression was measured in NAc cells from GAD_67_^+^ mice with the same treatment as immunofluorescence. Using trypsin-EDTA (Sigma), the cell disassociation solution for flow cytometry analysis was prepared, with cell counts conducted at 37°C after treatment. Subsequently, the cells underwent fixation in a solution of 0.5% paraformaldehyde in PBS and were incubated with antibodies specific for Rab10 and GFP at 4°C for overnight. Incubation of the cells was carried out for 2 h at room temperature with fluorescence-conjugated secondary antibodies (Alexa 568 and Alexa 488, 1:400, Invitrogen). Cell analysis was conducted using an LSR II Flow Cytometer from BD Biosciences (San Jose, CA) with FACSDiva software.

### 2.12 Immunoendocytosis analysis and image processing

Immunoendocytosis analysis was conducted as previously described (Terunuma et al., 2010; Meng et al., 2018). In accordance with methods described in other research, NAc cells derived from wild-type and *Rab10*-deficient mice were subjected to transfection of MYC-GABA _B1_R with the help of calcium phosphate (Jiang and Chen, 2006). The living cells were incubated with MYC antibodies in the culture medium for 15 min at 37°C. They were then washed and placed into a hypotonic solution (a 1:1 mix of Dulbecco’s modified Eagle’s medium and water) for 5 min at 37°C. Next, they were placed in an isotonic medium devoid of KCl for half an hour at 37°C. The living cells were subjected to no treatment or a 5-min exposure to cocaine. Then, the cells underwent fixation and were processed for immunofluorescence. A 10% solution of goat serum in PBS was applied for 60 min to prevent non-specific binding. Prior to permeabilization, GABA_B1_R’s membrane expression was visualized by MYC and secondary antibodies labeled with Alexa 568 (Invitrogen) at a 1:400 dilution. After permeabilization, the internalized receptors were visualized using secondary antibodies labeled with Alexa 488 (Invitrogen) at a 1:400 dilution. Employing the same method as immunofluorescence, confocal images were acquired to quantify fluorescence intensities. The maximum-intensity images from several stacks were acquired and processed using Fiji software.

### 2.13 pHluorin activity analysis and image processing

NAc cells from *Rab10*-deficient and wild-type mice were transfected with GABA_B1_R-pHluorin using calcium phosphate (Fu et al., 2020). For the elimination of fluorescent spots, the somas of neurons expressing pHluorin were subjected to 470 nm pulsed blue light stimuli at 2 Hz, with each pulse lasting 5 ms. Each experiment consisted of 10 pulses with 1-min intervals between them. Then, imaging analysis was immediately performed. Using the NIKON FN1 Confocal Microscope (NIR Apo 40x DIC Water N.A. 0.8), the fluorescence dynamics of GABA _B1_R-pHluorin (excitation 488 nm; EM: 500–550 nm) were captured, with images recorded at 1-s intervals and processed using Fiji software.

### 2.14 Electrophysiology analysis

Cultured NAc cells were moved to a recording chamber for continuous perfusion at a rate of 2–4 mL/min with oxygenated ACSF, with a composition of (in mM) 125 NaCl, 2.5 KCl, 25 NaHCO_3_, 1.25 NaH_2_PO_4_, 1.3 MgCl_2_, 2.5 CaCl_2_, and 11 glucose at 30°C. For visualization, a ×40 water-immersion objective on an upright fluorescence microscope (BX51WI; Olympus), complete with infrared-differential interference contrast video microscopy and epifluorescence (Olympus), was utilized. The patch pipettes, exhibiting a resistance of 4–6 M Ω, were crafted from G150TF-4 borosilicate glass (Warner Instruments). These pipettes were loaded with an internal solution comprising (in mM) 1 EGTA, 130 CsCl, 0.2 NaGTP, 2 MgATP, 10 HEPES, and 0.1% neurobiotin, pH 7.35, with an osmolarity ranging from 270 to 285 mOsm.

The internal solution for the measurement of mIPSCs included (in mM) 10 BAPTA, 115 potassium methylsulfate, 20 NaCl, 1.5 MgCl_2_, 10 sodium phosphocreatine, 0.4 NaGTP, and 4 MgATP, at pH 7.35 and osmolarity of 285 mOsm. To block AMPA, GABA_A_, and NMDA receptors, respectively, recordings were made in the presence of 20 µM CNQX (6-cyano-7-nitroquinoxaline-2,3-dione, MCE), 50 µM picrotoxin (Sigma), and 50 µM D-AP5. To isolate monosynaptic inputs, 1 μM tetrodotoxin (TTX, MCE) was added to the bath solution for inhibiting voltage-gated sodium channels (Petreanu et al., 2009). Recordings of electrophysiological signals were conducted at a temperature of 32°C using a MultiClamp 700B Amplifier. The signals were digitized using a Digidata 1440A Digitizer, sampled at a frequency of 10 kHz and filtered at 2 kHz. Data acquisition was carried out using pCLAMP software from Molecular Devices. mIPSCs were recorded at −70 mV. Regarding the pharmacological experiments, drugs were applied in the bath for durations ranging from 5 to 10 min. This included 50 μM picrotoxin for the inhibition of GABA_A_ receptor-mediated currents and 100 μM baclofen from Sigma to activate GABA_B_ receptors. Prior to baseline recording, cells were given 3 min to equilibrate after achieving the whole-cell configuration. Baseline responses were recorded for 5 min. Clampfit (Molecular Devices) and Mini Analysis Program (Synaptosoft) were used to analyze electrophysiological data. Access resistance was <25 MΩ, and cells exhibiting excessive fluctuations in access resistance (>20%) were not included in the analysis. The detailed parameter settings are consistent with those reported in the study conducted by Yang et al. (2018). Each recording lasted for 5 min, and a random 60-s segment was extracted from each for data analysis. Each experiment yielded six valid data points, and the same experiment was conducted three times. Given that the GAD_67_-GFP knock-in mice in this study, together with the previously published literature (Meredith, 1999), have demonstrated that over 90% of the NAc cells are GABAergic neurons, no steps were taken to identify the cell type in the electrophysiological experiments.

### 2.15 Measurement of Ca^2+^ influx

After isolation and plating, the NAc cells were prepared for Ca^2+^ analysis with the GCaMP system through calcium phosphate-mediated transfection, a method described by Jiang and Chen (2006). After NAc cells were treated with saline, cocaine (1 μM, 5 min post treatments), and/or baclofen (100 μM, 30 min post-treatments), cells in 48-well plates were live-imaged after 50 mM KCl treatments using a Nikon FN1 Confocal Microscope (NIR Apo 40× DIC Water, N.A. 0.8). High K^+^ elicited Ca^2+^ influx by depolarizing the cells’ membrane potential. The images were analyzed for the fluorescence dynamic of GCaMP (excitation 488 nm; EM: 500–550 nm). The images underwent processing using Rainbow RGB of Fiji software to create pseudo-color images. Relative fluorescence intensity is represented as ΔF/F, with F indicating the initial fluorescence after treatments with high K^+^ (50 mM KCl) and ΔF, indicating the fluorescence change after treatments with high K^+^ combined with cocaine and/or baclofen.

### 2.16 Statistical analysis

SPSS 20.0 and GraphPad Prism 6 (GraphPad software) were used for statistical analysis. To evaluate statistical differences, parametric statistical tests, including unpaired student’s *t*-tests, one-way analysis of variance (ANOVA), two-way ANOVA, or mixed-effects models, were used. Once a substantial main effect was identified via ANOVA, Bonferroni’s *post hoc* test or Dunnett’s *post hoc* analysis was employed. Dunnett’s multiple comparison test was employed to compare multiple experimental groups with a single control group, and Bonferroni’s multiple comparison test was applied for pairwise comparisons among multiple groups. Statistical significance was set as *p* < 0.05. Data are presented as the mean ± SEM.

## 3 Results

### 3.1 Rab10 expression in NAc regions was changed after cocaine treatment using transcriptomics and Western blot analysis

**FIGURE 1:**
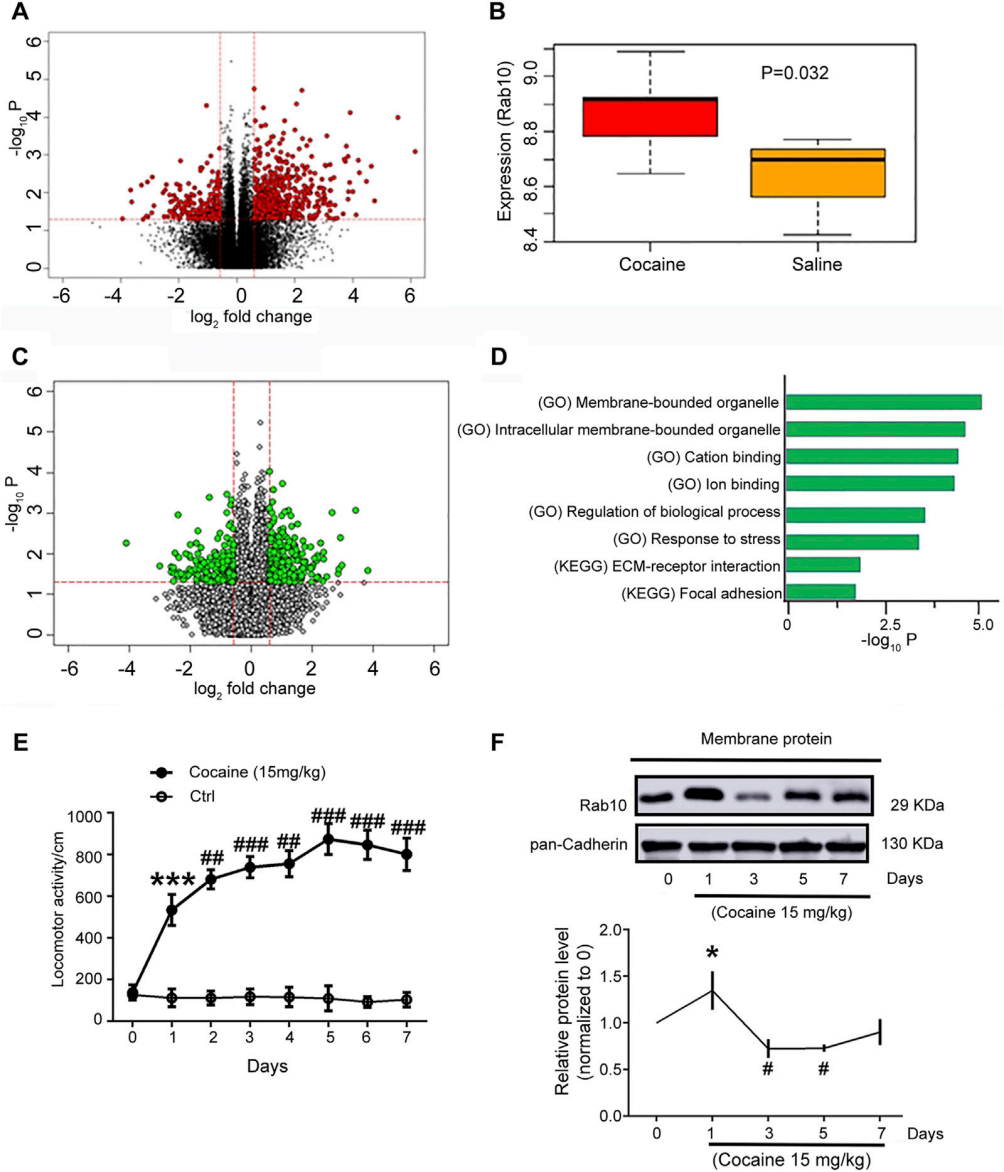
*Rab10* expression in NAc regions was changed after cocaine treatment. **(A)** Genes with a significant change in cocaine and saline groups (red dots). **(B)** *Rab10* transcript levels in NAc in cocaine and saline groups. **(C)** Genes with a significant change in groups with high and low expression of Rab10 (green dots). **(D)** Representative significantly enriched pathways. **(E)** Total distance traveled for 10 min by rats treated with saline or cocaine (15 mg/kg) in the open-field test on 7 consecutive days (n = 6). Data were presented as the means ± SEM. ***p < 0.001 compared with the saline group; ##*p* < 0.01 and ###*p* < 0.001 compared with the cocaine-treated group on day 1. **(F)** Rab10 expression measured on days 0, 1, 3, 5, and 7 after cocaine treatment (n = 3). Quantification of the relative protein level of Rab10 versus pan-Cadherin and normalization versus day 0. Data were presented as the means ± SEM. *p < 0.05 compared with cocaine-treated group on day 0; #*p* < 0.05 compared with cocaine-treated group on day 0.

In order to understand how Rab10 in the NAc regions may participate in response to cocaine treatment, we first sought to determine differences in its expression patterns with or without cocaine treatment. We therefore examined previously generated RNAseq data from mouse NAc treated with cocaine or saline (GEO dataset GSE18751; unpaired *t*-tests; false discovery rate (FDR) *p* < 0.05 and |fold change| > 1.5; Figure 1A) and found that *Rab10* was transcribed at significantly higher levels in the NAc of the cocaine-group compared to the control group (Figure 1B, *p* = 0.032). Further examination of the RNAseq data to identify genes that are more likely to affect cocaine addiction revealed 367 differentially expressed genes between *Rab10* high- and low-expression groups (unpaired *t*-tests; FDR *p* < 0.05 and |fold change| > 1.5; Figure 1C). By performing functional- and pathway-based enrichment analysis for those genes, we found 56 significantly enriched pathways (Figure 1D). Among these pathways, membrane-bounded organelle and cation-binding function suggested that membrane binding could contribute, along with Rab10, to the role of cocaine-associated behavioral effects.

The repeated cocaine administration at a dosage of 15 mg/kg led to hyperlocomotion on day 1, induced behavioral sensitization on day 3, and peaked on day 5 (Figure 1E), which was consistent with previously published findings (Peng et al., 2014; Hu et al., 2015; Xiong et al., 2018). Based on the results of *Rab10* induction under cocaine treatment, we next used Western blot analysis to measure the expression of Rab10 on the plasma membrane of rat NAc tissue sampled on days 0, 1, 3, 5, and 7 of cocaine treatment. We found that Rab10 exhibited significant, time-dependent changes in its membrane expression levels during exposure to cocaine (Figure 1F). The expression levels of Rab10 were generally elevated within day 1 of initiating the cocaine treatment regimen, which was consistent with the results of differential expression analysis. However, Rab10 levels subsequently decreased in the following time points, suggesting that it may play different roles in early (1 day and earlier) and late (1 day later) responses (i.e., acute versus chronic responses) to cocaine treatment.

### 3.2 *Rab10* deficiency in NAc regions inhibited cocaine-induced hyperlocomotion and behavioral sensitization, accompanied by an increase in the membrane expression of GABA_B_R

**FIGURE 2:**
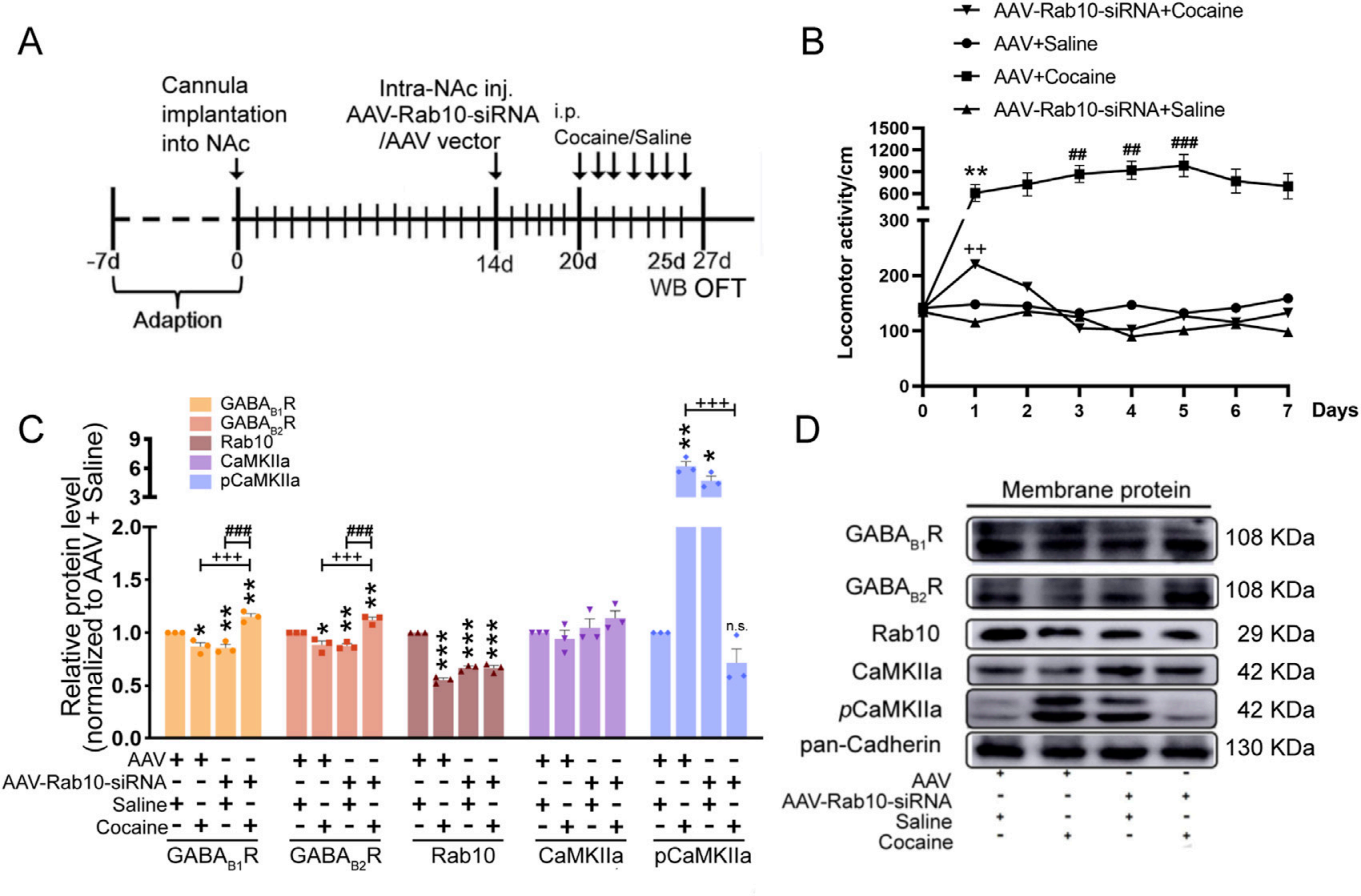
*Rab10* deficiency in NAc regions inhibited cocaine-induced behavioral sensitization by increasing the plasma membrane expression of GABA_B_R. **(A)** Experimental timeline of the cocaine-induced behavioral sensitization in *Rab10* deficiency rats. **(B)** Measurement of the total traveling distance in the open-field test (n = 6). Data were presented as the means ± SEM. **p < 0.01 compared with the AAV + saline group; ##*p* < 0.01 and ###*p* < 0.001 compared with AAV + cocaine-treated group on day 1; ++*p* < 0.01 compared with the AAV + cocaine group. **(C)** Quantification of the relative protein level of GABA_B1_R, GABA_B2_R, Rab10, CaMKIIa, and *p*CaMKIIa versus pan-Cadherin and normalization versus AAV + saline group (n = 3). Data were presented as the means ± SEM. *p < 0.05, **p < 0.01 and ***p < 0.001 compared with the AAV + saline group; +++*p* < 0.001 compared with the AAV + cocaine group; ###*p* < 0.001 compared with AAV-Rab10-siRNA + saline groups; n.s., no significance. **(D)** Representative plasma membrane protein Western blot of GABA_B1_R, GABA_B2_R, CaMKIIa, *p*CaMKIIa, and Rab10 obtained at 5 days post-treatment.

To determine whether Rab10 is related to behavioral effects induced by cocaine, we generated the AAV-Rab10-siRNA-GFP construct, which was microinjected into rat NAc regions 7 days before the administration of saline or cocaine (Figure 2A). With the extremely high density of Nissl bodies observed around the NAc shell, the position of the implanted cannula within the NAc was inferred through Nissl staining (Supplementary Figure S1A). Membrane expression of Rab10 decreased for 7, 10, and 14 days, with its lowest expression at 7 days (Supplementary Figure S1B).

After infection, data collected in the OFT for 7 successive days indicated that after saline treatment, rats expressing AAV-Rab10-siRNA showed similar locomotor activity compared to AAV-vehicle rats (Figure 2B, Table 1), suggesting that *Rab10* deficiency alone did not significantly impact locomotor activity under saline-treated physiological circumstances. In rats treated with AAV vehicles, cocaine administration significantly elevated locomotor activity and induced behavioral sensitization since day 3 (Figure 2B, Table 1). Moreover, in the cocaine-administered groups, the expression of AAV-Rab10-siRNA markedly reduced cocaine-elevated locomotor activity, and the development of behavioral sensitization was effectively blocked (Figure 2B, Table 1), indicating that *Rab10* deficiency could inhibit cocaine-elicited hyperlocomotion and behavioral sensitization.

**TABLE 1:** Statistical analyses of Figure 2B.

Test method	Comparison group	Degree of freedom and F value	*P*-value
One-way ANOVA and Dunnett’s *post hoc* test	Day 2 vs. Day 1 in AAV + cocaine group	F (6,35) = 5.650	0.5002
	Day 3 vs. Day 1 in AAV + cocaine group	F (6,35) = 5.650	0.0084
	Day 4 vs. Day 1 in AAV + cocaine group	F (6,35) = 5.650	0.0014
	Day 5 vs. Day 1 in AAV + cocaine group	F (6,35) = 5.650	0.0000
	Day 6 vs. Day 1 in AAV + cocaine group	F (6,35) = 5.650	0.2177
	Day 7 vs. Day 1 in AAV + cocaine group	F (6,35) = 5.650	0.7257

Test method	Comparison group	Degree of freedom and F value	*p-*value
Two-way ANOVA and Bonferroni’s *post hoc* test	AAV-Rab10-siRNA + cocaine vs. AAV + cocaine in Day 0	F (7,63) = 21.80	0.9999
	AAV-Rab10-siRNA + cocaine vs. AAV + cocaine in Day 1	F (7,63) = 21.80	0.0016
	AAV-Rab10-siRNA + cocaine vs. AAV + cocaine in Day 2	F (7,63) = 21.80	0.0007
	AAV-Rab10-siRNA + cocaine vs. AAV + cocaine in Day 3	F (7,63) = 21.80	0.0000
	AAV-Rab10-siRNA + cocaine vs. AAV + cocaine in Day 4	F (7,63) = 21.80	0.0000
	AAV-Rab10-siRNA + cocaine vs. AAV + cocaine in Day 5	F (7,63) = 21.80	0.0001
	AAV-Rab10-siRNA + cocaine vs. AAV + cocaine in Day 6	F (7,63) = 21.80	0.0011
	AAV-Rab10-siRNA + cocaine vs. AAV + cocaine in Day 7	F (7,63) = 21.80	0.0021

Test method	Comparison group	Degree of freedom and F value	*p-*value
Two-way ANOVA and Bonferroni’s *post hoc* test	AAV + cocaine vs. AAV + saline in Day 0	F (7,56) = 17.35	0.9999
	AAV + cocaine vs. AAV + saline in Day 1	F (7,56) = 17.35	0.0013
	AAV + cocaine vs. AAV + saline in Day 2	F (7,56) = 17.35	0.0018
	AAV + cocaine vs. AAV + saline in Day 3	F (7,56) = 17.35	0.0000
	AAV + cocaine vs. AAV + saline in Day 4	F (7,56) = 17.35	0.0000
	AAV + cocaine vs. AAV + saline in Day 5	F (7,56) = 17.35	0.0002
	AAV + cocaine vs. AAV + saline in Day 6	F (7,56) = 17.35	0.0018
	AAV + cocaine vs. AAV + saline in Day 7	F (7,56) = 17.35	0.0045

Our earlier findings indicated that cocaine inhibited the membrane expression of GABA_B_R in NAc regions by enhancing the phosphorylated calcium/calmodulin-dependent protein kinase II (*p*CaMKII–GABA_B1_R interaction) (Lu et al., 2023). Thus, after 5 days of cocaine treatment, we collected samples for Western blot analysis and analyzed the membrane expression of GABA_B_R, CaMKII, and *p*CaMKII in NAc neurons transfected with AAV-Rab10-siRNA. We found significantly lower membrane protein levels of GABA_B1_R and GABA_B2_R and the significantly higher protein level of *p*CaMKII induced in the NAc neurons transfected with AAV-Rab10-siRNA than those in AAV-vehicle neurons (Figures 2C, D). However, during cocaine treatments, the membrane expression of GABA_B1_R and GABA_B2_R was significantly increased, along with the decrease in *p*CaMKII, in the neurons transfected with AAV-Rab10-siRNA compared to that in neurons transfected with the AAV vector control (Figures 2C, D). These differential responses suggested that Rab10 could modulate the GABA_B_R membrane expression of NAc neurons during cocaine-induced hyperlocomotion and behavioral sensitization.

### 3.3 Rab10 expression was decreased in cocaine-treated *GAD*_*67*_^*+*^ NAc neurons

**FIGURE 3:**
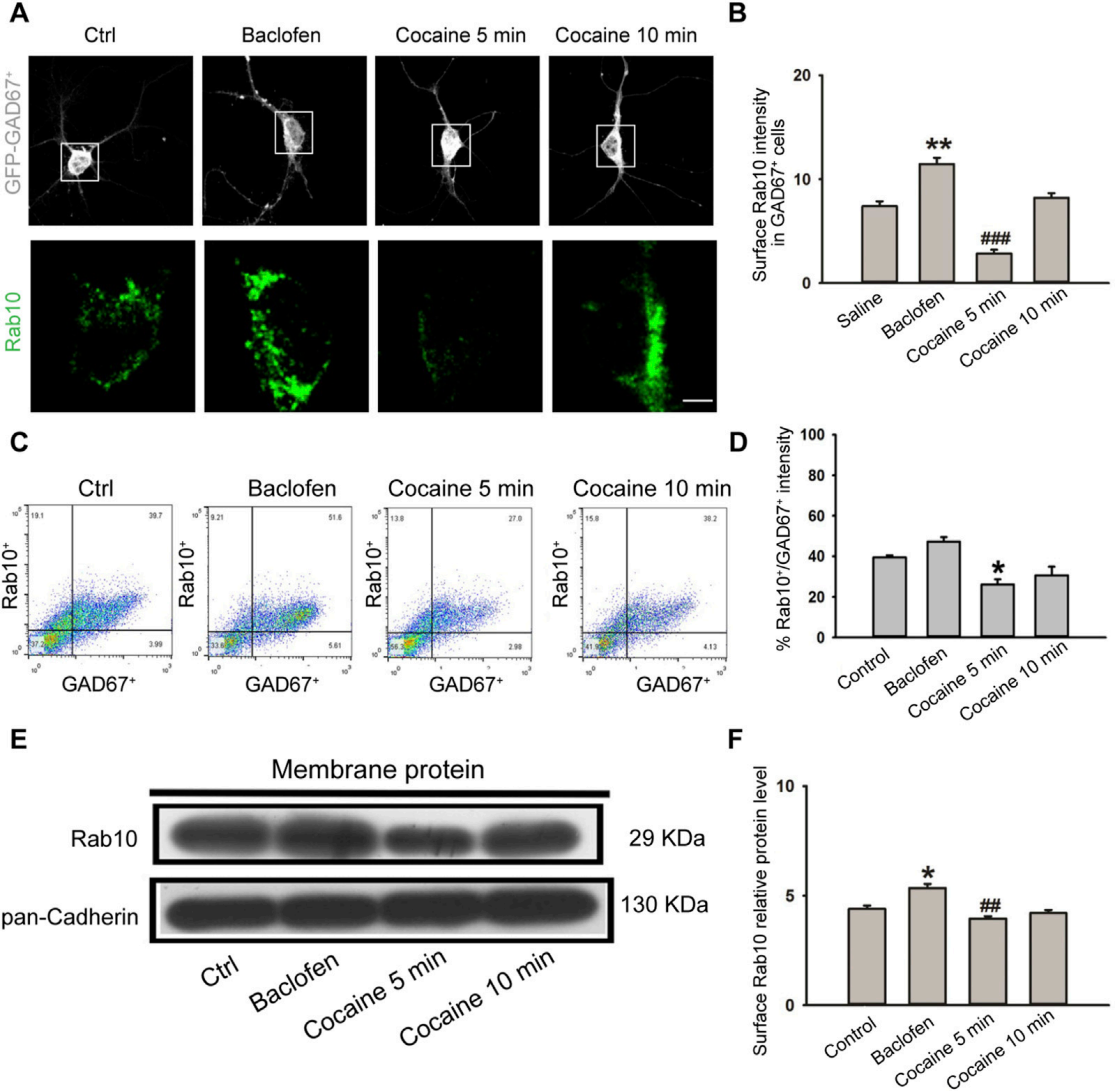
Cocaine treatment inhibited plasma membrane expression of Rab10 in NAc *GAD*_*67*_^*+*^ cells. **(A)** NAc *GAD*_*67*_^*+*^ cells were stained with antibodies against Rab10 (green) and GFP (gray). Scale bar, 10 μm. Cells were treated with saline, baclofen, or cocaine. **(B)** Data from **(A)** were presented as the means ± SEM (n = 3). ###*p* < 0.001 and **p < 0.01 compared with the saline group. **(C)** Dot plot analysis of *GAD*_*67*_^*+*^ neurons from NAc. **(D)** Relative fluorescence intensity of Rab10^+^/*GAD*_*67*_^*+*^ (n = 3). Data were presented as the means ± SEM. *p < 0.05 compared with the control group. **(E)** Representative plasma membrane protein Western blot of Rab10 obtained after treatment with saline, baclofen, or cocaine. **(F)** Quantification of the relative protein level of Rab10 versus pan-Cadherin and normalization versus saline (n = 3). ##*p* < 0.01 and *p < 0.05 compared with the saline group.

*In vivo* experiments have shown that cocaine treatment can affect the expression of Rab10, and the effect varies depending on the time of treatment. Next, we explored the effect of cocaine treatment on the expression of Rab10 in NAc *GAD*_*67*_^*+*^ neurons *in vitro*. We isolated *GAD*_*67*_^*+*^ neurons from the NAc of GAD_67_-GFP knock-in mice and treated them in culture with cocaine (1 μM). We then examined Rab10 expression at 5 and 10 min of cocaine exposure. Immunofluorescent labeling with confocal microscopy (Figures 3A, B) and flow cytometers (Figures 3C, D) and Western blot analysis (Figures 3E, F) revealed significantly decreased Rab10 membrane fluorescence, as well as decreased protein levels of Rab10. At the same time, the treatment of the GABA_B_R agonist baclofen led to a high induction of Rab10 expression. Based on these data, we suggested that Rab10 likely regulates the membrane expression of GABA_B_R.

### 3.4 *Rab10* deficiency in cocaine-treated NAc neurons led to higher GABA_B_R membrane expression

**FIGURE 4:**
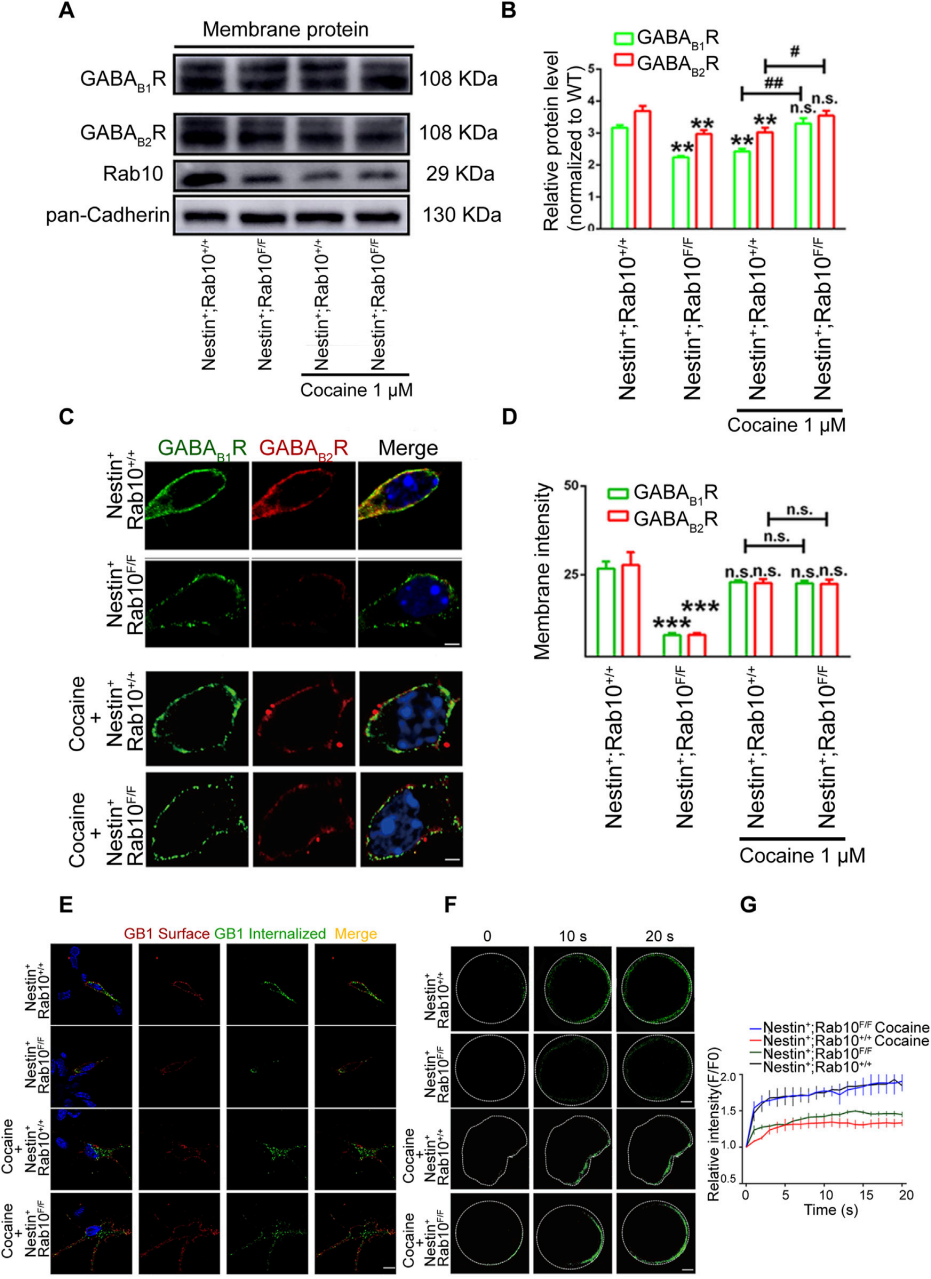
Differential GABA_B_R plasma membrane expression in NAc neurons from *Rab10* deficiency mice under cocaine treatment. **(A)** Representative plasma membrane protein Western blot of Rab10, GABA_B1_R, and GABA_B2_R in NAc cells from wild-type (WT, Nestin+; *Rab10* ^+/+^) or *Rab10*-deficient (WT, Nestin+; *Rab10* ^*F/F*^) mice after saline or cocaine treatment. Data were presented as the means ± SEM. **(B)** Quantification of the relative protein level of GABA_B_R and Rab10 versus pan-Cadherin (n = 3). Data were presented as the means ± SEM. **p < 0.01 compared with the wild-type (WT, Nestin+; *Rab10* ^+/+^) with saline treatment; ##*p* < 0.01 and #*p* < 0.05 compared with wild-type (WT, Nestin+; *Rab10* ^+/+^) with cocaine treatment. **(C)** NAc cells were stained with antibodies against GABA_B1_R (green), GABA_B2_R (red), and DAPI (blue). Scale bar, 10 μm. **(D)** Data were presented as the means ± SEM (n = 3). ***p < 0.001; n.s., no significance. **(E)** NAc neurons were stained against surface GABA_B1_R (red) and internalized GABA_B1_R (green). Scale bar, 10 μm. **(F)** NAc neurons transfected with GABA_B1_R-pHluorin were stimulated with ∼470 nm laser, followed by time-lapse imaging. White dashed line: cell membrane. Scale bar, 10 μm. **(G)** Normalized fluorescence intensity of GABA_B1_R-pHluorin.

We next investigated whether and how Rab10 contributes to the membrane expression of GABA_B_R. Western blot (Figures 4A, B) and immunostaining (Figures 4C, D) showed that membrane levels of GABA_B1_R and GABA_B2_R were both significantly lower in the NAc neurons in *Rab10*-deficient mice than in WT mice. Immunoendocytosis showed that GABA_B_R appeared to accumulate throughout the cells of the WT mice, including the plasma membrane and perinuclear regions (Figure 4E). Moreover, in *Rab10*-deficient mice, GABA_B1_R internalization was observed at the plasma membrane, while the internalized GABA_B_R is located in the cytoplasmic edges (Figure 4E). Furthermore, a decrease in the GABA_B1_R-pHluorin signal (Figures 4F, G) could be observed in the NAc neurons of *Rab10*-deficient mice compared to that in WT mice. These findings indicated that Rab10 could serve as a regulator of GABA_B_R membrane expression.

In the cocaine-treated group, although the fluorescence levels of GABA_B1_R and GABA_B2_R were not significantly higher in the NAc neurons of *Rab10*-deficient mice compared to WT mice (Figures 4C, D), an obvious increase in the expression of GABA_B1_R and GABA_B2_R protein was identified by Western blot analysis (Figures 4A, B). Immunoendocytosis data showed that under cocaine treatment, the WT mice showed GABA_B1_R internalization at the plasma membrane, with the majority of internalized GABA_B_R remaining spatially restricted in the proximity of the plasma membrane (Figure 4E). However, in *Rab10*-deficient mice, GABA_B_R appeared to accumulate throughout the whole cells, including the plasma membrane and perinuclear regions (Figure 4E). Moreover, in cocaine-treated NAc neurons of *Rab10*-deficient mice, an increase in GABA_B1_R–pHluorin signals could be observed (Figures 4F, G). These results indicated that Rab10 could inhibit the membrane expression of GABA_B_R in cocaine-treated neurons and further suggested differences in the NAc neurons’ response to saline-associated homeostasis or cocaine-associated allostasis.

### 3.5 *Rab10* deficiency in cocaine-treated NAc neurons promoted the inhibitory function of GABA_B_R

**FIGURE 5:**
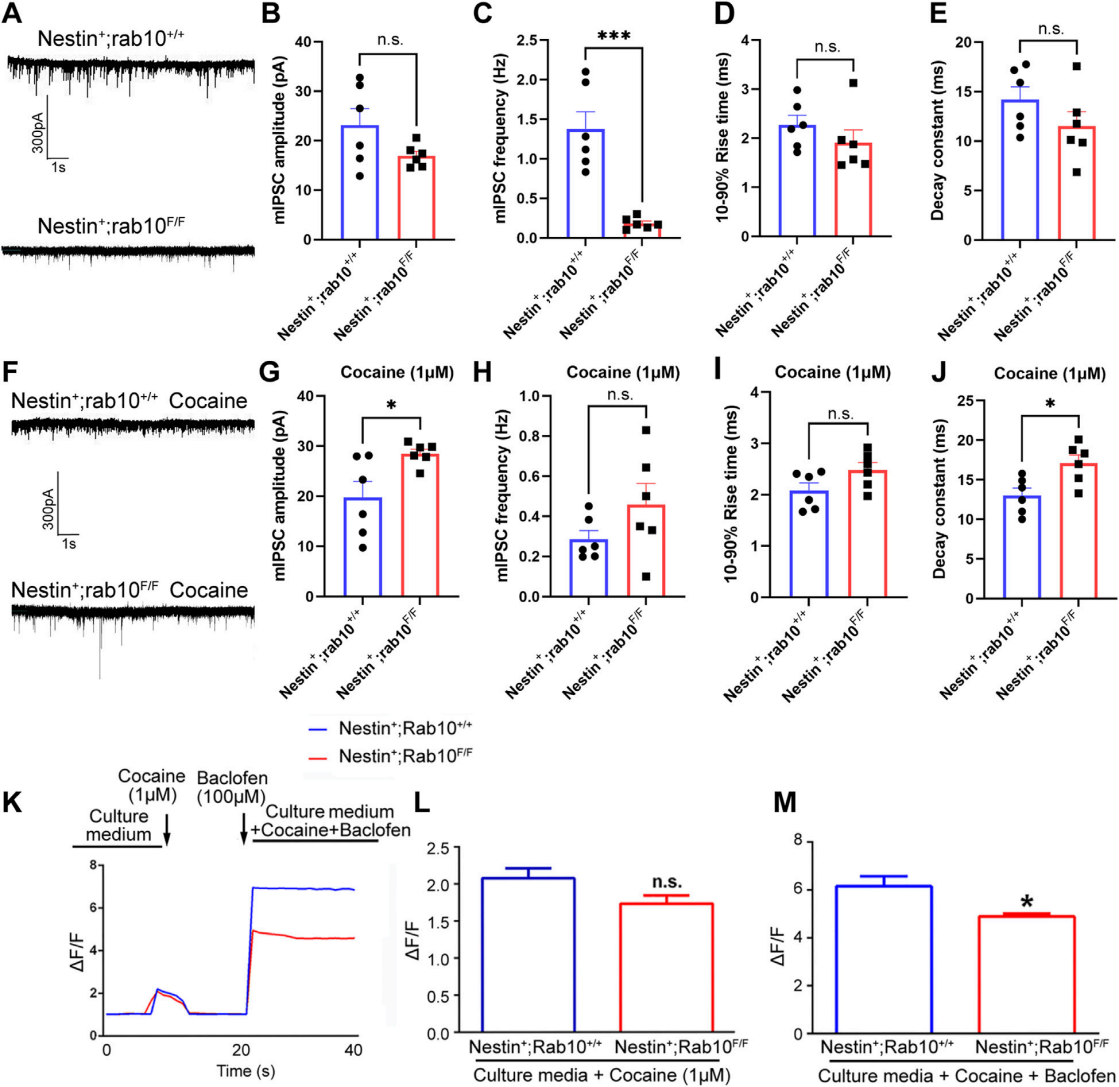
mIPSC recordings were used to measure GABA_B_R function in NAc cells from wild-type and *Rab10*-deficient mice. **(A)** mIPSC record of wild-type (WT, Nestin+; *Rab10* ^+/+^) and Rab10-deficient (Nestin+; *Rab10* ^*F/F*^) mice at −70 mV under saline treatment. **(B, C)** Quantification of the mIPSC amplitude and frequency. **(D)** 10%–90% rise time. **(E)** Decay constant. **(F)** mIPSC record of wild-type (WT, Nestin+; *Rab10* ^+/+^) and Rab10-deficient (Nestin+; *Rab10* ^*F/F*^) mice at −70 mV under cocaine treatment. **(G, H)** Quantification of the mIPSC amplitude and frequency recorded under cocaine treatment. **(I)** 10%–90% rise time. **(J)** Decay constant. **(K)** Real-time measurement of the GCaMP6 fluorescence intensity in NAc neurons, following cocaine and/or baclofen treatment. **(L)** Bar graphs quantifying the GCaMP6 fluorescence intensity in NAc neurons following cocaine treatment. **(M)** Bar graphs quantifying the GCaMP6 fluorescence intensity in NAc neurons following cocaine + baclofen treatment. Data were presented as the means ± SEM. *p < 0.05; ***p < 0.001; n.s., no significance.

We recorded mIPSCs to measure GABA_B_R function in NAc from wild-type (WT, Nestin^+^; *Rab10* ^+/+^) and *Rab10*-deficient (Nestin^+^; *Rab*10^F/F^) mice. NAc cells were categorized into four groups based on the absence or presence of Rab10 knockout and cocaine treatment. The frequency of mIPSCs was significantly lower in *Rab10*-deficient mice than in WT mice (Figures 5A, C), but *Rab10* deficiency did not affect the amplitude and dynamic properties of mIPSCs (Figures 5B, D, E). To further characterize the effects of *Rab10* deficiency on GABA_B_R *in vitro*, we conducted patch-clamp recordings of mIPSCs in NAc from WT and *Rab10*-deficient mice. The results indicated that the amplitude and decay constant of mIPSCs were significantly higher in the *Rab10*-deficient group than those of the control group (Figures 5F, G, J), indicating that under cocaine treatment, the deficiency of *Rab10* leads to an enhancement of neural inhibition at the postsynaptic area, which is consistent with the increased membrane expression of GABA_B_R observed in our Western blotting and immunoendocytosis. The frequency and 10%–90% rise time showed no significant change (Figures 5H, I).

To further determine the impact of *Rab10* deficiency under cocaine treatment on the function of GABA_B_R, we measured changes in GCaMP6 fluorescence in these WT and *Rab10*-deficient neurons treated with cocaine combined with baclofen. The results showed that cocaine could increase Ca^2+^-influx evoked by high K^+^ in both WT and *Rab10*-deficient mouse NAc, and no significant difference was observed between the two groups (Figures 5K, L). These experiments thus demonstrated that cocaine amplifies the Ca^2+^ influx. Furthermore, baclofen-treated *Rab10*-deficient neurons exposed to cocaine exhibited a substantially lower Ca^2+^ influx than WT neurons (Figures 5K, M). These results thus demonstrated that Rab10 is a key positive regulator of the baclofen-amplified Ca^2+^-influx process in cells treated with cocaine.

## 4 Discussion

**FIGURE 6:**
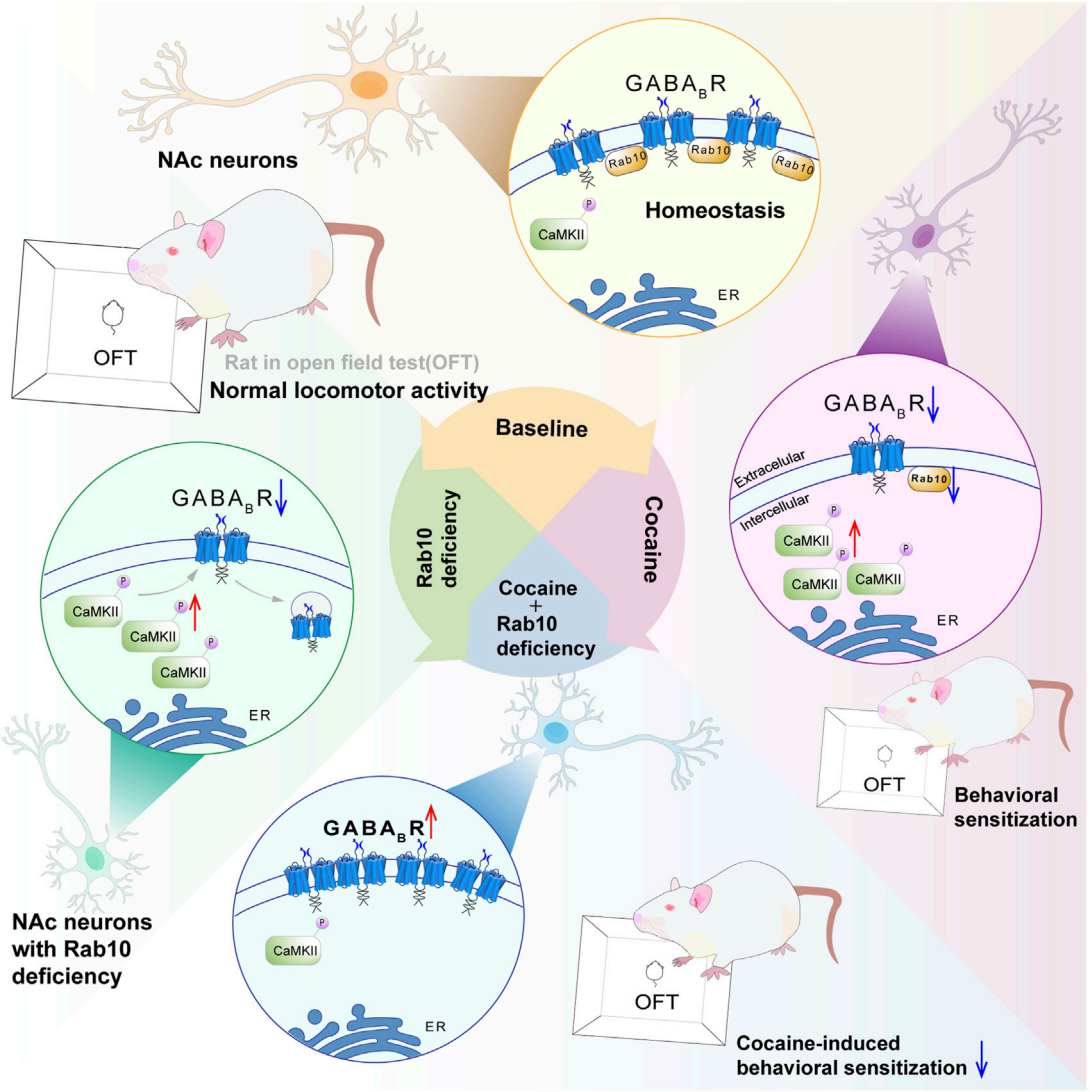
Proposed model of Rab10 action in cocaine-induced behavioral sensitization by altering GABA_B_R expression. Rab10 is a key regulator of GABA_B_R plasma membrane expression in the NAc region under basal state or after cocaine treatment. *Rab10* deficiency increased plasma membrane expression of GABA_B_R in NAc neurons of cocaine-treated animals to the physiological level, which, in turn, inhibited cocaine-induced behavioral sensitization measured by an open-field test.

Previous proteomics studies have shown that repeated treatment with methamphetamine can inhibit Rab10 expression in rat neostriatal membranes. Conversely, inhibiting neuronal Rab10 activity in *D. melanogaster* resulted in decreased effects of methamphetamine and caffeine treatment (Vanderwerf et al., 2015). However, the expression patterns of Rab10 during cocaine treatment and its potential effects (and mechanisms underlying these effects) on cocaine addiction remain largely unexplored. In this study, our results suggest that Rab10 participates in cocaine-induced behavioral effects (see Figure 6 for the model). This conclusion is reinforced by several pieces of evidence: 1) Western blot analysis indicated that Rab10 was significantly upregulated and subsequently downregulated during repeated response to cocaine; 2) most importantly, NAc neuron-specific genetic ablation of *Rab10* inhibited cocaine-induced hyperlocomotion and behavioral sensitization; and 3) cocaine treatment on NAc *GAD*_*67*_^*+*^ neurons *in vitro* decreased the expression of Rab10. Furthermore, we demonstrated that Rab10 could serve as a regulator of GABA_B_R membrane expression. Given that several studies have suggested that cocaine-induced behavioral effects are closely related to GABA_B_ receptors (Jayaram and Steketee, 2004; Lhuillier et al., 2007) and our previous study also showed that the CaMKII-mediated phosphorylation of GABA_B_ receptors within the nucleus accumbens is closely related to cocaine-induced behavioral sensitization (Lu et al., 2023), we propose that the effects of Rab10 on cocaine-induced behavioral effects are associated with GABA_B_ receptor membrane expression in NAc.

*In vitro* experiments of this study have revealed that Rab10 exerts significant regulatory effects on the membrane expression of GABA_B_R. To further elucidate this regulatory effect, we conducted electrophysiological experiments. The mIPSCs we examined, particularly their amplitudes, reflected the expression and function of postsynaptic GABA_B_R. Under cocaine treatment, the absence of *Rab10* significantly increased the amplitude of mIPSCs, suggesting that the deficiency of *Rab10* under this condition can enhance the expression and function of postsynaptic GABA_B_R. GABA is widely recognized as an inhibitory neurotransmitter, and the activation of GABA_B_R has been reported to reduce locomotor activity and alleviate cocaine-induced hyperlocomotion and behavioral sensitization (Lhuillier et al., 2007). Therefore, the results of our *in vivo* experiments, which indicated that the absence of *Rab10* reduces cocaine-induced behavioral effects, are consistent with the results of our series of *in vitro* experiments, suggesting that the role of Rab10 in cocaine-induced behavioral effects is associated with GABA_B_R membrane expression in NAc. Given the presence of potential compensatory mechanisms *in vivo*, results obtained from *in vitro* experiments may sometimes exhibit inconsistencies with those from *in vivo* studies. Therefore, further *in vivo* experiments are warranted to confirm the mechanism we propose in this study. In electrophysiological experiments, we also observed changes in the frequency of mIPSCs, suggesting that the role of Rab10 may also be associated with presynaptic effects. Considering that GABA_B_R is expressed at both presynaptic and postsynaptic sites (Gerrard et al., 2018), this presynaptic effect may also be related to the regulation of GABA_B_R by Rab10 at the presynaptic level, which requires further investigation. Rab10 has been widely reported to have impacts on behavioral activity. In this work, we demonstrated that under physiological conditions, *Rab10* deficiency had no marked effect on locomotor activity. In rats, acute cocaine treatment induces increased locomotor activity, and chronic cocaine exposure leads to behavioral sensitization, which is considered a result of neuroadaptive changes (Robinson and Berridge, 2008). Under acute cocaine treatment, the knockdown of *Rab10* led to a decrease in locomotor activity, demonstrating that Rab10 influences hyperlocomotion induced by cocaine. Under chronic cocaine treatment, the initiation of Rab10’s regulation of GABA_B_R membrane expression resulted in the involvement of GABA_B_R in the process by which Rab10 affects cocaine-induced hyperlocomotion and behavioral sensitization. In this study, the knockdown of *Rab10* blocked both hyperlocomotion and behavioral sensitization induced by cocaine. However, the absence of behavioral sensitization could not only be attributed to the deficiency of *Rab10* but might also be due to the suppression of locomotor activity. In the OFT of another study, although normally reducing *Rab10* expression resulted in a slight increase in motor activity, which may be attributed to increased interest in contextual novelty, their overall results are consistent with our findings (Bunner et al., 2023). However, the knockdown of *Rab10* in dopaminergic neurons is reported to completely reverse the Parkinson’s related bradykinesia caused by *LRRK2-G2019S*, indicating that the knockdown of *Rab10* can promote behavioral activity (Fellgett et al., 2021). In contrast, our data showed that *Rab10* deficiency could inhibit cocaine-induced hyperlocomotion and locomotor sensitization. This discrepancy may be attributed to differences in experimental animals (i.e., rats and flies; cocaine-induced behavioral sensitization and *LRRK2-G2019S*-induced bradykinesia). Furthermore, given that we microinjected AAV-Rab10-siRNA-GFP construct into rats’ NAc region to lower the Rab10 level, while a previous study used CRISPR/Cas9, *Rab10* ^RNAi^, and deGradFP techniques, the different methods of Rab10 reduction could also be a possible reason.

In the context of psychostimulant-induced addiction, the principal mechanism underlying the “long-loop” GABAergic feedback is the activation of GABA_B_ receptors in the NAc neurons (Edwards et al., 2017; Yang et al., 2018). Furthermore, it has been demonstrated that cocaine inhibits the GABA_B_R-dependent function of NAc neurons *in vivo* (Edwards et al., 2017; Torregrossa et al., 2019). Self-administration of methamphetamine inhibits GABA_B_R-dependent responses (Padgett et al., 2012; Munoz et al., 2016). The selective GABA_B_R agonist baclofen is reported to reduce dopamine efflux into the NAc triggered by cocaine, accompanied by a significant reduction in psychostimulant-induced self-administration, conditioned place preference, and hyperlocomotion (Hotsenpiller and Wolf, 2003; Brebner et al., 2005; Fuchs et al., 2008). Moreover, baclofen attenuates motivational disturbances such as drug craving, drug-seeking behavior, and relapse, as observed in clinical and preclinical investigations (Hotsenpiller and Wolf, 2003).

Among the more than 60 reported mammalian Rab proteins, several have been shown to regulate GABA_B_R membrane availability, in addition to the characterization of Rab10 presented in this study. For example, GABA_B_R localizes to Rab7-positive lysosomes as well as Rab4-or Rab11-positive recycling endosomes under normal physiological conditions (Terunuma et al., 2010; Kantamneni et al., 2014; Zemoura et al., 2016; Zemoura et al., 2019; Li et al., 2020), and increased levels of GABA_B_R are accompanied by decreases in Rab7 and Rab11 (Zemoura et al., 2019). Overexpression of Rab7 results in considerably enhanced protein accumulation of GABA_B_R, not restricted to membrane expression (Zemoura et al., 2016). In addition, a chemically induced increase in GABA_B_R membrane expression during long term potentiation (LTP) can be prevented by blocking Rab4 recycling (Kantamneni et al., 2014). Rab5 was also detected through mass spectrometry analysis of proteins that co-immunoprecipitated with GABA_B_R (Li et al., 2020). Previous studies have suggested that Rab10 plays an important role in the directional membrane insertion underlying axon and dendrite development (Xu et al., 2014; Taylor et al., 2015). Its reported role in regulating receptor trafficking, including the low-density lipoprotein receptor (Khan et al., 2022; Hamilton et al., 2023), growth factor receptor (Rivero-Ríos et al., 2020), Toll-like receptor (Wang et al., 2010), transferrin receptor (Brewer et al., 2016), suggests that Rab10 may also participate in GABA_B_R vesicle trafficking in the central nervous system, such as its internalization, degradation, or export to the endoplasmic reticulum. It is also demonstrated to play a role in the sorting and retrograde axonal transport of internalized TrkB receptors (Lazo and Schiavo, 2023). In this study, we also suggested that Rab10 can regulate the expression of GABA_B_R, which may be a possible mechanism of cocaine’s effect. The classic mechanism by which cocaine exerts its biological effects is through the blockade of the dopamine transporter (Volkow et al., 1997). However, our *in vitro* experiments were carried out in cultured NAc cells, which do not have the dopamine transporter. Therefore, in our *in vitro* experiments, the reduction of GABA_B_R induced by cocaine is not achieved through the conventional mechanism. Based on our research, we hypothesize that Rab10 may be a potential target for cocaine, and cocaine may regulate the expression of GABA_B_R by modulating Rab10. Specifically, cocaine treatment leads to a reduction in the expression of Rab10, which in turn results in decreased expression of GABA_B_R. In summary, our findings demonstrate an important role of Rab10 in regulating cocaine-induced behavioral effects, which is closely related to GABA_B_R membrane expression. Further investigations are required to confirm whether Rab10 exerts its effects on cocaine-induced behavioral effects through the regulation of membrane expression and function of GABA_B_R, as indicated by the *in vitro* experiments conducted in this study, and determine whether and which Rab10-associated proteins are responsible for GABA_B_R membrane expression and signal transduction. Since it has been reported that Rab10 can regulate trafficking of multiple receptors, such as the epidermal growth factor receptor (Rivero-Ríos et al., 2020), Toll-like receptor (Wang et al., 2010), and transferrin receptor (Brewer et al., 2016), the potential for Rab10-GABA_B_R signaling as a treatment target could be constrained by notable off-target effects.

## Data Availability

The raw data supporting the conclusions of this article will be made available by the authors, without undue reservation.
