# Supplementary material for: Role of Rab10 in cocaine-induced behavioral effects is associated with GABAB receptor membrane expression in the nucleus accumbens
Source: Front Pharmacol. 2024 Nov 27;15:1496657. doi: 10.3389/fphar.2024.1496657 (PMC11635607; doi:10.3389/fphar.2024.1496657)
Supplement: Supplementary file 1 [file DataSheet1.DOCX]

Supplementary Material


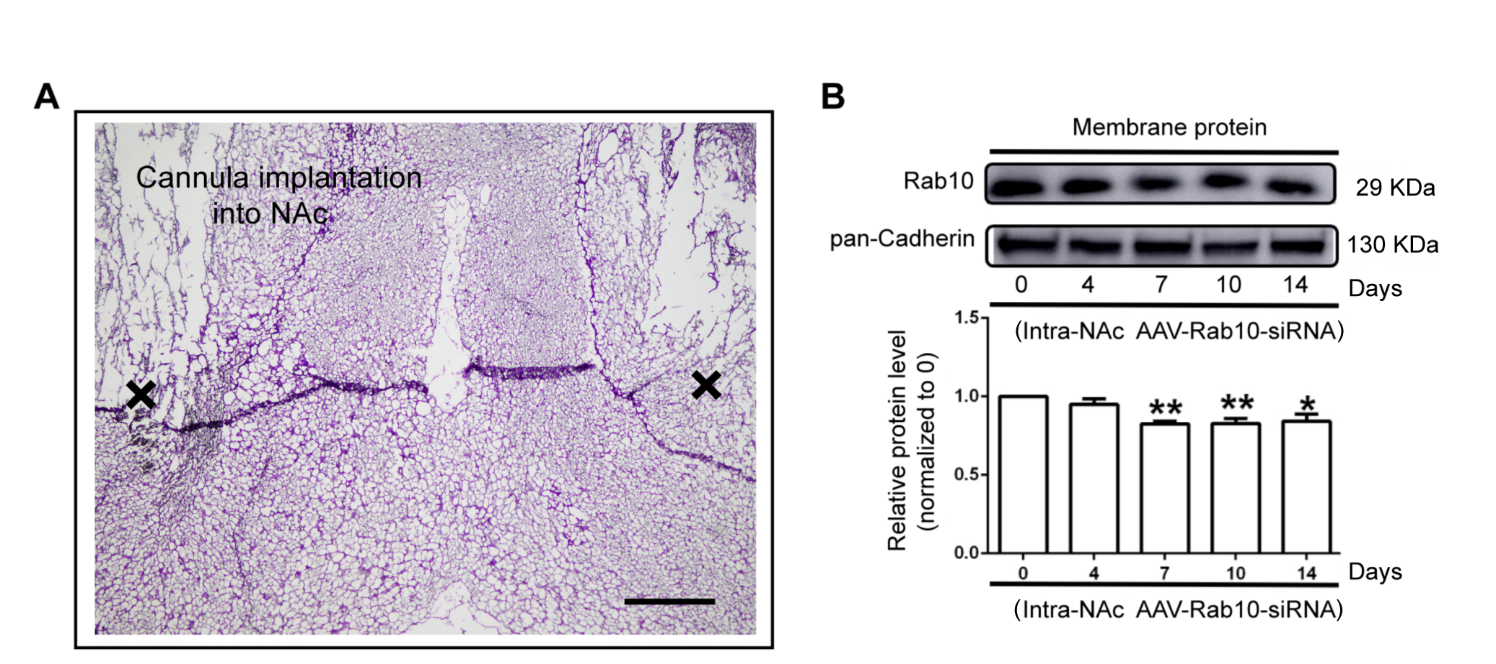


**Supplementary Figure 1.** Virus-mediated gene transfer decreased Rab10 expression, with its lowest expression at 7 days. **A** Nissl staining of NAc tissues showing the cannula implantation site. Scale bar, 250 μm. The black cross marked the evident Nissl bodies within the NAc and suggested sufficient protein synthesis. **B** Representative plasma membrane protein Western blot of Rab10 obtained at 0, 4, 7, 10, and 14 days post infection. Bar graph showing the quantification of the relative protein level of Rab10 versus pan-Cadherin and normalization versus 0 day. Data were presented as means ± SEM. **P* < 0.05, ***P* < 0.01, one-way ANOVA and Dunnett’s post-hoc test.
